# Top-Emitting Active-Matrix Quantum Dot Light-Emitting Diode Array with Optical Microcavity for Micro QLED Display

**DOI:** 10.3390/nano12152683

**Published:** 2022-08-04

**Authors:** Kuo-Yang Lai, Shuan Yang, Tung-Chang Tsai, I-An Yao, Chiu-Lien Yang, Chih-Ching Chang, Hsueh-Shih Chen

**Affiliations:** 1 Ph.D. Program in Prospective Functional Materials Industry, College of Engineering, National Tsing Hua University, Hsinchu 30013, Taiwan; 2 Department of Materials Science and Engineering, National Tsing Hua University, Hsinchu 30013, Taiwan; 3 Technology Development Division, Innolux Corporation, Miaoli 35053, Taiwan; 4 Hsinlight Inc., South Campus, National Tsing Hua University, Hsinchu 30013, Taiwan

**Keywords:** active matrix, AMQLED, ITO/Ag/ITO, microcavity, QD, top emission

## Abstract

An electroluminescent quantum-dot light-emitting diode (QLED) device and a micro QLED device array with a top-emitting structure were demonstrated in this study. The QLED device was fabricated in the normal structure of [ITO/Ag/ITO anode]/PEDOT:PSS/PVK/QDs/[ZnO nanoparticles]/Ag/MoO_3_, in which the semi-transparent MoO_3_-capped Ag cathode and the reflective ITO/metal/ITO (IMI) anode were designed to form an optical microcavity. Compared with conventional bottom-emitting QLED, the microcavity-based top-emitting QLED possessed enhanced optical properties, e.g., ~500% luminance, ~300% current efficiency, and a narrower bandwidth. A 1.49 inch micro QLED panel with 86,400 top-emitting QLED devices in two different sizes (17 × 78 μm^2^ and 74 × 40.5 μm^2^) on a low-temperature polysilicon (LTPS) backplane was also fabricated, demonstrating the top-emitting QLED with microcavity as a promising structure in future micro display applications.

## 1. Introduction

Colloidal quantum dots (QDs) have attracted significant attention in various applications, such as energy harvesting, sensing, and displays, because of their irreplaceable optical properties such as high quantum yield (QY), wide absorption range, tunable emission wavelength, and high-purity color [1,2,3,4]. For liquid crystal displays (LCDs), QDs can improve their color gamut by simply introducing QDs in various application forms, including QD films [5,6,7], QD light guide plates [8], QD color converters [9,10,11], and QD-converted light-emitting diodes (QD-LEDs) [12,13,14,15,16,17]. A QD-enhanced LCD can be called a QD-LCD. On the other hand, electroluminescence (EL)-based QD light-emitting diodes (QLEDs) [18,19,20,21,22,23,24] can work without a backlight and may be called a self-emitting QLED or EL-QLED. A self-emitting QLED has excellent color performance, such as narrow and adjustable emission bandwidth and wavelength, viewed as next-generation lighting and display technology. Compared with other displays, QLED has higher image contrast and a faster response than QD-LCD [25], as well as a broader color gamut coverage than organic light-emitting diode (OLED) or micro-LED [26,27,28]. Furthermore, the solution-based fabrication process of QLED offers the advantages of large-area applications at a low manufacturing cost.

Both passive-matrix (PM) and active-matrix (AM) driving electrode systems may be used to realize display applications, where the latter is beneficial for a larger display area or higher resolution. In terms of AM-based QLED, the conventional device design is bottom-emitting, where the light emission is extracted from the glass substrate side [29,30,31,32]. However, the opening of the bottom-emitting device and the light intensity are limited by the opaque thin-film transistor (TFT) circuit in the AM backplane. Hence, a higher operating current density is required to produce sufficient display brightness, which consumes more energy and reduces the operation lifetime of QLED devices. Instead, the top-emitting structure is more suitable for a high-resolution AMQLED display [33]. In the previous studies on top-emitting QLED (TEQLED), a semi-transparent metal electrode (Ag) served as the top electrode, allowing light to pass through it, while Al or Ag was employed as a reflective bottom electrode that increased light extraction [33,34,35,36]. Ag has a high reflectance and a low absorbance in the visible wavelength range, and it has been commonly utilized as a bottom electrode that simultaneously reflects light to the top side. However, Ag has a low work function (~4.3 eV) and is not beneficial to hole injection in the normal device structure [34,37]. Furthermore, the reflective bottom electrode and the semi-transparent top electrode may form an optical microcavity, significantly affecting the light coupling and interference in the devices [34,38,39,40]. Therefore, the optical microcavity effect associated with the thickness of each device layer should be considered in TEQLEDs.

This study demonstrates a high-resolution monochrome AMQLED with a top-emitting structure using a conventional low-temperature polysilicon (LTPS) backplane used for OLEDs. The TEQLED is fabricated in a normal structure, where an ITO/Ag/ITO (IAI) multilayer electrode acts as a reflective bottom anode and a thin Ag film capped with MoO_3_ serves as the semitransparent top cathode. By adjusting the thickness of the ZnO layer and QD layer to form an optical microcavity, TEQLEDs can exhibit fivefold the maximum luminance (~129,200 cd·m^−2^) and threefold higher current efficiency (~28.4 cd·A^−1^) than bottom-emitting QLED (BEQLED).

## 2. Experimental Section

### 2.1. Fabrication of TEQLED

ITO/Ag/ITO-coated glass substrate was ultrasonically cleaned in DI water, acetone, and IPA for 15 min sequentially. Then, the substrate was treated by UV ozone to modify the wettability and the work function. The PEDOT:PSS solution (AI 4083) was filtered using a 0.45 μm Nylon filter before being spin-coated on a substrate at 3000 rpm for 50 s. The substrate was transferred to a nitrogen-filled glovebox and annealed at 140 °C for 20 min. PVK (Mw 25,000–50,000 g·mol^−1^, 5 mg·mL^−1^ in toluene) was spin-coated onto HIL and annealed at 140 °C for 20 min, resulting in a 17 nm thick HTL. The QDs (G-QD-1, 0.5 nm shell thickness, wavelength 529 nm/FWHM 34.1 nm/PLQY 80%/EL-QD 1-low driving voltage, provided from Hsinlight Inc., Taiwan) was spin-coated at 2000 rpm for 40 s and annealed at 80 °C for 10 min; the concentration of QD solution was adjusted from 5 mg·mL^−1^ to 20 mg·mL^−1^ for the desired thickness. ZnO NPs (in IPA) were spin-coated at 2000 rpm for 40 s and annealed at 60 °C for 10 min, the concentration of ZnO solution was adjusted from 10 mg·mL^−1^ to 30 mg·mL^−1^ for the desired thickness. Finally, the semi-transparent cathode consisting of Ag (30 nm) and MoO_3_ (30 nm) was thermally deposited under a high vacuum (<8 × 10^−6^ torr). A cover glass and the UV-curable resin were used to encapsulate the device. The synthesis of ZnO nanocrystals was described in our previous paper [41]. For comparison, devices prepared with two other alloyed ZnCdSeS QDs with different shell thicknesses (G-QD-2/1.1 nm shell thickness/wavelength 523 nm/FWHM 20.5 nm/PLQY 65% and G-QD-3/1.6 nm shell thickness/wavelength 521 nm/FWHM 17.7 nm) were also fabricated using the same processes and parameters. The ligand type of all QDs was oleic acid.

### 2.2. Characterizations

Photoluminescence (PL) spectra and electroluminescence (EL) spectra were measured by a spectrofluorometer (Edinburgh FS5 from England). Optical absorption was measured by a UV/Vis/NIR spectrophotometer (Jasco V-770 from Japan). Surface morphology of the cathode was observed by field-emission scanning electron microscopy (FESEM, Hitachi SU8010 from Japan). Cross-sectional transmission electron microscopy (TEM) images and the line-scan profile of energy-dispersive X-ray spectroscopy were acquired on a high-resolution TEM (HRTEM, JEOL JEM-F200 from Japan). The device cross-section sample was prepared with a focused-ion-beam (FIB) system (SII NANOTECH SMI2050 from Japan) equipped with a Ga^+^ ion beam. The valence band maximum (VBM) of QDs was identified by ultraviolet photoelectron spectroscopy (UPS, ULVAC-PHI PHI 5000 Versaprobe II from Japan) with a UV source (He I α, 21.22 eV). The Tauc plot derived from the absorption spectrum was used to analyze the optical bandgap of QDs. The current density–voltage–luminance characteristics of QLEDs were measured by a source meter unit (SMU, Keithley 2450 from USA) and a luminance meter (TOPCON BM-9A from Japan). The contact angle of water on Ag and ITO was acquired from a contact angle analyzer (FTA125 from USA).

## 3. Results and Discussion

### 3.1. Selection of Appropriate Type of QDs for AMQLED Array and Panel

**Figure 1:**
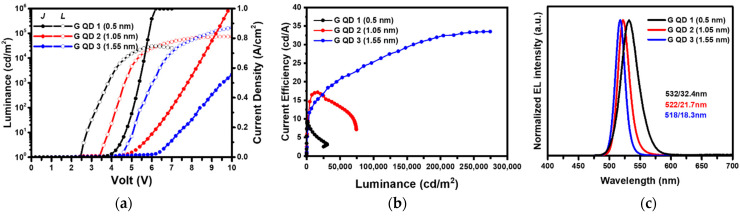
(**a**) *J*–*V*–*L* and (**b**) *CE*–*L* characteristics and (**c**) EL spectra of the BEQLEDs prepared from G-QDs with different shell thickness. All the devices were prepared using the same device process and the BEQLED structure, i.e., ITO/PEDOT:PSS (40 nm)/PVK (17 nm)/QDs (25 nm)/ZnO (20 nm)/Al (100 nm). In (**b**), the device with G-QD-3 should possess a maximum luminance more 280,000 cd·m^−2^, which is the detection limit of the luminance meter.

To operate a QLED device array, the QLED driving conditions, such as the turn-on voltage and current–voltage relation, are much more important than for a single QLED device. Hence, the selection of a proper type of QDs is one of the keys. Otherwise, the QLED device array may not correctly work. For a QLED device array applied to a display panel, the driving condition and display quality (e.g., wavelength, bandwidth, and performance) of every QLED device are significantly influenced by the external circuit and driver. In terms of AMQLED, a conventional OLED display driving system can be utilized to drive an AMQLED panel, whereby the QD emitting layer may necessarily be similar to the organic emitting layer in terms of device properties. In general, a lower driving voltage (<5 V) is required for existing AM LTPS-TFT backplanes to drive every emitting device. Three types of green alloyed ZnCdSeS QDs with different shell thicknesses (0.5 nm/G-QD-1, 1.1 nm/G-QD-2, and 1.6 nm/G-QD-3, Figure S1) were first fabricated in the conventional BEQLED structure to evaluate their applicability. Figure 1 shows the current density–voltage–luminescence and current efficiency–luminance (*CE*–*L*) characteristics, as well as the EL spectra, of devices with G-QD-1, G-QD-2, and G-QD-3. The turn-on voltage (*V*_on_) increased from 2.5 V to 4.6 V with increasing shell thickness from 0.5 nm to 1.6 nm, indicating that carrier injection was more efficient for the thinner-shelled QDs. With thicker-shelled QDs, the devices showed higher current efficiency and luminance, representing better carrier confinement at the high electric field contributed by the thick shell. In particular, the device with G-QD-3 possessed the maximum luminance and a current efficiency of over 280,000 cd·m^−2^ and 33 cd·A^−1^, respectively. Although thick-shelled QDs may have a higher device performance, a high operating voltage range is not suitable for the current LTPS-TFT backplanes. Therefore, G-QD-1 was used to study the device and panel properties of AMQLED display panels.

### 3.2. QLED with the Top-Emission Structure

**Figure 2:**
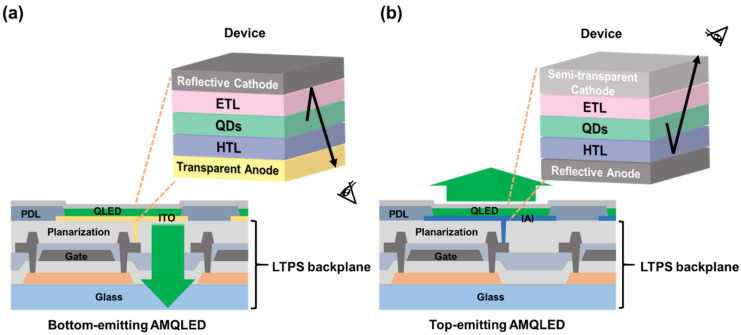
(**a**) AMQLED with a bottom-emitting structure. The light emission intensity is determined by the aperture ratio restricted by the pixel driving circuit. (**b**) AMQLED has a top-emitting structure, which offers a larger opening and, thus, more efficient emission than the bottom-emitting one.

Conventional QLED devices are generally fabricated in a so-called normal device structure with bottom emission, e.g., glass substrate/ITO anode/hole-transporting layer (HTL)/QDs/electron-transporting layer (ETL)/metal cathode, in which the emitting light can be extracted from the glass substrate side. For an AM display panel with a bottom-emitting structure, the ITO anode is prepared on a TFT backplane to control every single emitting device (i.e., pixel). Therefore, the emission intensity of pixels is restricted by the opening space (also called the aperture size) of the TFT backplane involving electrodes and wiring, limiting the pixel size and display resolution, as schematically illustrated in Figure 2a. On the other hand, the top-emitting device structure has a larger aperture size, allowing a higher emission intensity, which is more suitable for a higher-resolution AM-based QLED display panel (e.g., >150 ppi), as shown in Figure 2b. With a top-emission design, the light generated by the QLED devices passes through the top cathode side; hence, the bottom anode must be reflective to increase the light extraction efficiency.

**Figure 3:**
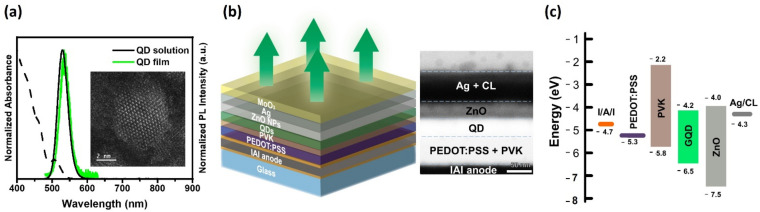
(**a**) The normalized PL and absorption spectra of QDs in the solution form (octane) and film form (spin-coated on a glass). The inset shows a TEM image of a QD (5.8 ± 0.8 nm). (**b**) Schematic diagram and cross-sectional TEM image of the TEQLED device structure in this study. (**c**) The energy level diagram of the TEQLED. The QD conduction band and valence band energies were estimated from UPS and optical absorption spectra. The values of the other layers were adopted from the literature [43,44].

Figure 3a shows the optical absorption and PL spectra of G-QD-1. The emission wavelength and full width at half maximum (FWHM) of the QDs in octane were 529 nm and 34.1 nm, respectively. The PL spectrum of the spin-coated QD film showed a red-shifted emission peak and a narrower FWHM (534 nm/32.4 nm) caused by Förster resonance energy transfer (FRET) in a densely packed QD film [42]. A cross-sectional TEM image of the top-emitting QLED (TEQLED), together with its schematic structure, is shown in Figure 3b. The TEQLED was fabricated in a normal structure, i.e., [ITO/Ag/ITO]/PEDOT:PSS/PVK/QDs/[ZnO NPs]/[semi-transparent Ag]/MoO_3_, where [ITO/Ag/ITO], PEDOT:PSS, PVK, QDs, ZnO NPs (nanoparticles), semi-transparent Ag, and MoO_3_ were the reflective anode, hole injection layer (HIL), HTL, emission layer (EML), ETL, cathode, and capping layer, respectively. Figure 3c shows the energy band diagram of the TEQLED, in which the QD energy level was estimated from the ultraviolet photoelectron spectroscopy (UPS) spectrum and Tauc plot (Figure S2).

### 3.3. Fabrication of Bottom Reflective Anode in the IAI Structure

**Figure 4:**
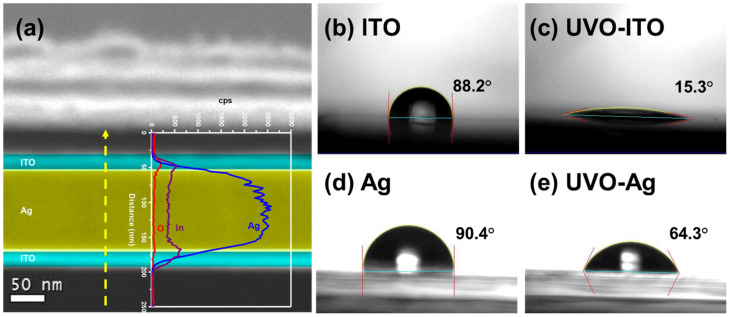
(**a**) The cross-sectional STEM image of ITO/Ag/ITO multilayer anode. The inset shows the corresponding EDS line scan profile. The contact angle of water on (**b**) ITO, (**c**) UVO-treated ITO, (**d**) Ag, and (**e**) UVO-treated Ag.

A pure Ag film is generally used as a bottom reflective electrode because of its high conductivity and reflectance. Nevertheless, the metal film is relatively less hydrophilic even after a surface modification. Instead, a tri-layered transparent conductive oxide/metal/transparent conductive oxide composite film, i.e., ITO/Ag/ITO (IAI) with a thickness of 20 nm/110 nm/20 nm, was adopted as the reflective anode (Figure 4a). Compared with the pure Ag film, the IAI anode has the advantages of better material stability and enhanced optical reflection because of ITO protection [45]. Moreover, the IAI anode has more surface processability. It can be feasibly treated with UV/ozone to ensure a better match in the work function and higher chemical compatibility with the subsequent HIL layer [23,46]. For example, the ITO surface of IAI became more hydrophilic after UV/ozone treatment compared with the UV/ozone-treated Ag film, which was still relatively more hydrophobic and could affect the film deposition of PEDOT:PSS (Figure 4b–e).

### 3.4. Top Ag Cathode with MoO_3_ Capping Layer

Thermally evaporated Ag film was prepared as the semi-transparent top cathode instead of sputtering-based transparent conductive oxides because the latter process may degrade the QD layer [47,48]. To prepare a transparent metal electrode, ultrathin (e.g., <10 nm) Ag is generally necessary. Unfortunately, Ag easily forms a discontinuous film or aggregate-like particulate morphology on a substrate, significantly affecting the optical properties (e.g., light scattering) and the carrier injection [49]. In the current QLED panels, a relatively thicker Ag cathode (~ 30 nm) was prepared to ensure that the device worked correctly because the TFT panels could not appropriately work with a thinner Ag film. However, a thicker Ag cathode could slightly reduce the optical transmittance of the QLED pixels. Moreover, a refractive index mismatch between the Ag cathode and the air decreased the device emission intensity. Consequently, an additional MoO_3_ capping layer was introduced to clad the Ag cathode to improve light extraction [50,51]. MoO_3_ has a high refractive index and a low extinction coefficient [52,53]. By thermally depositing a 30 nm MoO_3_ capping layer, the transmittance of Ag film could be increased (Figure S3). With the MoO_3_ capping layer, TEQLEDs showed an 81% improvement in the current efficiency (8.8 cd·A^−1^ to 15.9 cd·A^−1^, Figure S4). In addition, some current leakage in the TEQLED without a capping layer was observed, which could be explained by the poor Ag film quality (Figure S5). It has been reported that a MoO_3_ capping layer can improve the Ag film morphology and inhibit Ag grain agglomeration [54,55].

### 3.5. Design of Optical Microcavity for TEQLEDs

**Figure 5:**
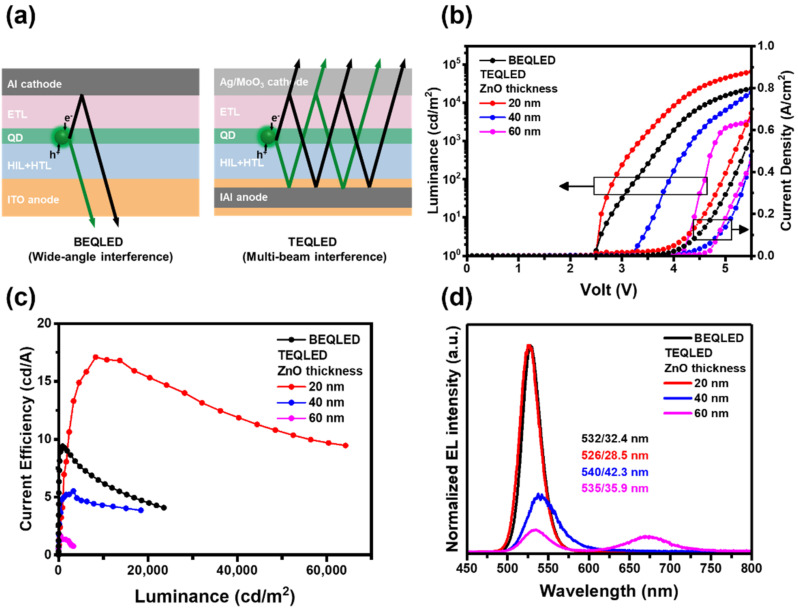
(**a**) Schematic diagram of the light interference modes in BEQLED and TEQLED. (**b**) *J*–*V*–*L* and (**c**) *CE*–*L* characteristics, and (**d**) EL spectra of the BEQLED and TEQLEDs with various ZnO thickness. The reference BEQLED consisted of ITO/PEDOT:PSS (40 nm)/PVK (17 nm)/QDs (25 nm)/ZnO (20 nm)/Al (100 nm). The TEQLED structure consisted of IAI anode/PEDOT:PSS (40 nm)/PVK (17 nm)/QDs (25 nm)/ZnO (x nm)/Ag (30 nm)/MoO_3_ (30 nm).

The optical length of top-emitting devices significantly affects their optical performance. Sandwiched by an IAI reflective anode and a semi-transparent Ag cathode, a microcavity structure was prepared in the TEQLEDs. Figure 5a schematically shows the two interference modes occurring in BEQLED and TEQLED, i.e., wide-angle and multi-beam interference. The constructive interference conditions are given below [56].

(1) $$m\times 2\pi =2\pi \frac{\sum {2n}_{i}d_{i}\cos\theta }{\lambda }-\left|\varphi_{\mathrm{anode}}\right|\;(\mathrm{wide}-\mathrm{angle}\;\mathrm{interference}),$$

(2) $$m\times 2\pi =2\pi \frac{\sum {2n}_{i}d_{i}\cos\theta }{\lambda }-\left|\varphi_{\mathrm{anode}}\right|-\left|\varphi_{\mathrm{cathode}}\right|\;(\mathrm{multi}-\mathrm{beam}\;\mathrm{interference}),$$

where n and d are the refractive index and the thickness of the layer, respectively. *φ* is the reflective phase shift of the electrode. The structure consists of a highly reflective back mirror electrode and a transparent output mirror electrode, forming an optical microcavity [57]. With an appropriate film thickness and tailored design, the microcavity can enhance the device brightness and the color purity, which is beneficial for energy-efficient displays with a high color gamut [58]. The resonant wavelength of the microcavity mainly depends on the optical length. If the QD emission wavelength meets the resonant wavelength of the microcavity, constructive interference can enhance the light emission intensity. Since the Ag/MoO_3_ cathode had higher reflectance than ITO, the enhancement in TEQLED was more significant. Table 1 shows a comparison of QLED devices prepared in the bottom-emission and top-emission structure.

To investigate the resonant wavelength of the microcavity, the device optical length was varied by the ZnO layer thickness, which could be feasibly adjusted [59]. Figure 5 gives the *J*–*V*–*L* and *CE*–*L* characteristics, as well as EL spectra, of TEQLEDs with 20, 40, and 60 nm ZnO thickness. The 20 nm ZnO TEQLED had the lowest *V*_on_ (~2.5 V), while the 40 nm ZnO and 60 nm ZnO TEQLEDs had a higher *V*_on_ of 3.2 V and 4.2 V, respectively (Figure 5b). In addition, the 20 nm ZnO TEQLED had the highest efficiency of 17 cd·A^−1^, but the device efficiency decreased with increasing ZnO thickness (Figure 5c). Figure 5d shows the EL spectra of TEQLEDs operated at 2 mA. The TEQLEDs with 20, 40, and 60 nm ZnO layers had an EL wavelength/FWHM of 526 nm/28.5 nm, 540 nm/42.3 nm, and 535 nm/35.9 nm, respectively. The 20 nm ZnO TEQLED had a shorter EL wavelength and narrower bandwidth compared with the PL spectra of QDs in either solution or film form (wavelength/FWHM = 529 nm/34.1 nm and 534 nm/32.4 nm, Figure 3a). This fact indicates that a proper design of microcavity resonance can improve the optical property of TEQLEDs.

However, the device with a 40 nm or 60 nm ZnO layer had a broader and red-shifted EL curve. For the device with a 60 nm ZnO layer, a deep-red emission band appeared at 675 nm, ascribed to the red emission of PVK amplified by the microcavity [60]. Accordingly, the inappropriate optical length may affect the device performance of TEQLEDs. Table 2 gives a summary of device data for the TEQLEDs with different ZnO thicknesses.

A control BEQLED sample was also fabricated with the structure of ITO/PEDOT:PSS (40 nm)/PVK (17 nm)/QDs (33 nm)/ZnO (20 nm)/Al (100 nm) for reference (black curves in Figure 5b–d). The *V*_on_ of the BEQLED was 2.5 V, corresponding to the optical bandgap of green QDs (2.34 eV). The low *V*_on_ value reveals that carriers could be smoothly injected into the QDs through the carrier transport material that was energy level-matched. The current efficiency of BEQLED showed a max value of 9.4 cd·A^−1^ at a luminance of 1000 cd·m^−2^. Furthermore, the EL spectrum of the BEQLED (wavelength/FWHM = 532 nm/32.4 nm) was somewhat similar to the PL spectrum of the QD film (wavelength/FWHM = 534 nm/32.4 nm, Figure 3a), suggesting that no optical resonance effect existed in the bottom-emission structure.

**Figure 6:**
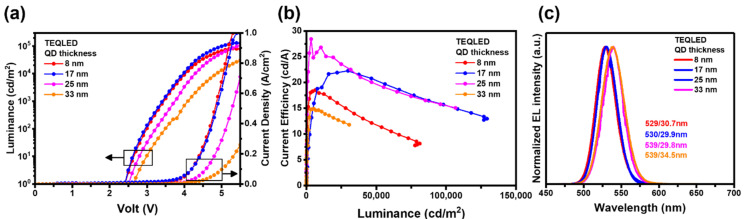
(**a**) *J*–*V*–*L* and (**b**) *CE-L* characteristics, and (**c**) EL spectra of TEQLED with different QD thickness. The structure of TEQLEDs was IAI anode/PEDOT:PSS (40 nm)/PVK (17 nm)/QDs (x nm)/ZnO (30 nm)/Ag (30 nm)/MoO_3_ (30 nm). In order to meet the display criteria in green wavelength, the ZnO thickness was increased to 30 nm to form a resonant wavelength close to ~534 nm.

The effect of the thickness of QD layer was also studied to improve the device properties of TEQLEDs. Devices with 30 nm ZnO were first prepared to form a resonant wavelength close to ~534 nm, meeting the display criteria of green wavelength. An increase in the QD thickness slightly raised the *V*_on_ from 2.5 V to 2.7 V and reduced the current density, as shown by the *J*–*V*–*L* characteristics in Figure 6a. The result may be explained by a thicker QD layer with a large amount of long alkyl chain ligands (i.e., oleic acid), leading to higher resistance in the device. For TEQLEDs with a 17 or 25 nm thick QD layer, the device luminance could reach over 100,000 cd·m^−2^, which is an approximately fivefold enhancement compared with BEQLED. As a result, the maximum current efficiencies of the TEQLEDs were enhanced to 20–30 cd·A^−1^ (Figure 6b). In addition, the FWHMs of TEQLEDs with 8, 17, and 25 nm thick QD layers were all narrower than those of BEQLED, indicating that the microcavity effect with constructive interference could also reduce the bandwidth and improve the color purity, as shown by the EL spectra in Figure 6c.

Moreover, when the thickness of QD layer was increased from 8 to 33 nm, the EL emission peak red-shifted from 529 to 539 nm, suggesting that the resonant wavelength of the cavity moved to a longer value because of increasing cavity length. The TEQLED with a 25 nm QD layer had the highest current efficiency of ~28 cd·A^−1^ and the max brightness of ~106,700 cd·m^−2^ at the EL wavelength of ~539 nm. The relatively low current efficiency of TEQLED with a 33 nm QD layer is attributable to the poor carrier injection and destructive interference in the microcavity structure. Table 3 summarizes the device performance and the QD thickness of TEQLEDs.

### 3.6. QLED Array Fabricated on a 1.49 Inch LTPS-TFT Substrate for Micro Display

**Figure 7:**
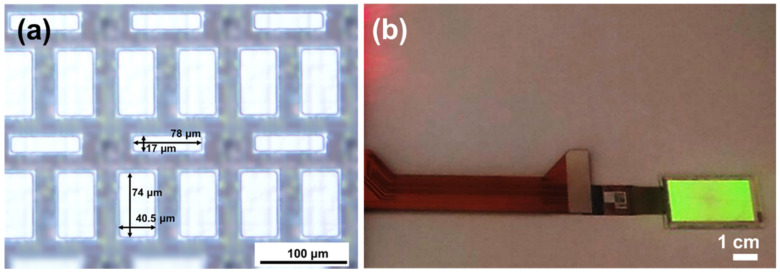
(**a**) Optical microscopic image of the AM TFT backplane employed in this study. The TFT backplane provided two different device sizes (17 × 78 μm^2^ and 74 × 40.5 μm^2^), as obtained from a commercial production line. (**b**) Photograph of 1.49 inch top-emitting AMQLED device array (number of device or sub-pixels = 86,400) operated at 5 V. An LTPS-TFT backplane with a 7T1C pixel circuit design was used to drive the AMQLED panel.

The top-emitting QLED structure was further fabricated into a micro device array to demonstrate its potential application in a micro display panel. A 1.49 inch TFT glass backplane with an aperture ratio of 36.8% was employed as a substrate, as shown in Figure 7a. The pixel-defining area of the backplane had two different dimensions (17 × 78 μm^2^ and 74 × 40.5 μm^2^). Fabrication of the micro QLED device array on the TFT backplane followed the same spin-coating-based process of a single QLED device. Figure 7b presents a fabricated QLED panel composed of 86,400 green top-emitting devices. The QLED panel could be operated in the range between 3 V and 5 V, exhibiting promise for application in a high-resolution micro monochrome QLED display.

## 4. Conclusions

A 1.49” top-emitting micro AMQLED array with an optical microcavity was demonstrated in this study. The QLED device was fabricated in the top-emitting normal structure of [ITO/Ag/ITO]/PEDOT:PSS/PVK/QDs/ZnO/[Ag-MoO_3_]. The MoO_3_-capped Ag cathode and the ITO/metal/ITO (IMI) anode formed an optical microcavity, enhancing the device performance. By adjusting the thickness of ZnO and QD layers to 30 nm and 25 nm, respectively, to optimize the optical microcavity, the luminance and the current efficiency of the top-emitting QLED could exceed 100,000 cd·m^−2^ and 28 cd·A^−1^, respectively, representing fivefold and threefold increases compared a conventional bottom-emitting QLED. The emission bandwidths of the devices were also reduced by 2 to 4 nm because of the microcavity effect. The AMQLED display panel with the current OLED LTPS-TFT backplane and driving system demonstrates significant potential for lighting and display applications in the near future.

## Figures and Tables

**Table 1:** Comparison of devices with the BEQLED and TEQLED structure.

	BEQLED	TEQLED
Aperture ratio	Low	High
Microcavity effect	Weak	Strong
Emission peak/FWHM	Depends on QD film	Sensitive to layer thickness

**Table 2:** Tuning of the optical length of cavity resonance by ZnO thickness for TEQLEDs. The reference device was a BEQLED without optimized structure.

TEQLED	*V*_on_ (V)	*L*_max_ (cd·m^−2^)	*CE*_max_ (cd·A^−1^)	EL (nm)
ZnO 20 nm	2.5	64,200	17.0	526/28.5
ZnO 40 nm	3.2	18,410	5.5	540/42.3
ZnO 60 nm	4.2	3,320	1.6	535/35.9
Ref. device	2.5	23,550	9.4	532/32.4

**Table 3:** Performance summary for the TEQLEDs with various QD thickness and 30 nm ZnO.

QD Thickness (nm)	*V*_on_ (V)	*L*_max_ (cd·m^−2^)	*CE*_max_ (cd·A^−1^)	EL (nm)
8	2.5	81,100	18.4	529/30.7
17	2.5	129,200	22.2	530/29.9
25	2.6	106,700	28.4	539/29.8
33	2.7	30,600	14.8	539/34.5

## Data Availability

Publicly available datasets were analyzed in this study.

## Supplementary Materials

The following supporting information can be downloaded at https://www.mdpi.com/article/10.3390/nano12152683/s1, Figure S1: TEM image of dried ZnCdSeS QD film, Figure S2: UPS spectrum and Tauc plot of the green QDs used in this work, Figure S3: The transmittance spectra and the reflectance spectra of Ag and Ag/capping layer with different ZnO NPs thickness, Figure S4: Current density-voltage-luminance characteristics and (b) Luminance-current efficiency characteristics for the TEQLED devices with or without a capping layer (thickness ~ 30 nm), Figure S5: The cross-sectional SEM images of films.
