# Supplementary material for: Top-Emitting Active-Matrix Quantum Dot Light-Emitting Diode Array with Optical Microcavity for Micro QLED Display
Source: Nanomaterials (Basel). 2022 Aug 4;12(15):2683. doi: 10.3390/nano12152683 (PMC9370811; doi:10.3390/nano12152683)
Supplement: Supplementary file 1 [file nanomaterials-12-02683-s001.zip › nanomaterials-1817700-supplementary.pdf]

nanomaterials

Supporting Information

Top-Emitting Active-Matrix Quantum Dot Light-Emitting Diode Array with Optical Microcavity for Micro QLED Display

Kuo-Yang Lai [1], Shuan Yang [2], Tung-Chang Tsai [3], I-An Yao [3], Chiu-Lien Yang [3], Chih-Ching Chang [4] and Hsueh-Shih Chen [1,*]

[1] Ph.D. Program in Prospective Functional Materials Industry, College of Engineering, National Tsing Hua University, Hsinchu 30013, Taiwan
[2] Department of Materials Science and Engineering, National Tsing Hua University, Hsinchu 30013, Taiwan
[3] Technology Development Division, Innolux Corporation, Miaoli 35053, Taiwan
[4] Hsinlight Inc., South Campus, National Tsing Hua University, Hsinchu 30013, Taiwan
* Correspondence: chenhs@mx.nthu.edu.tw

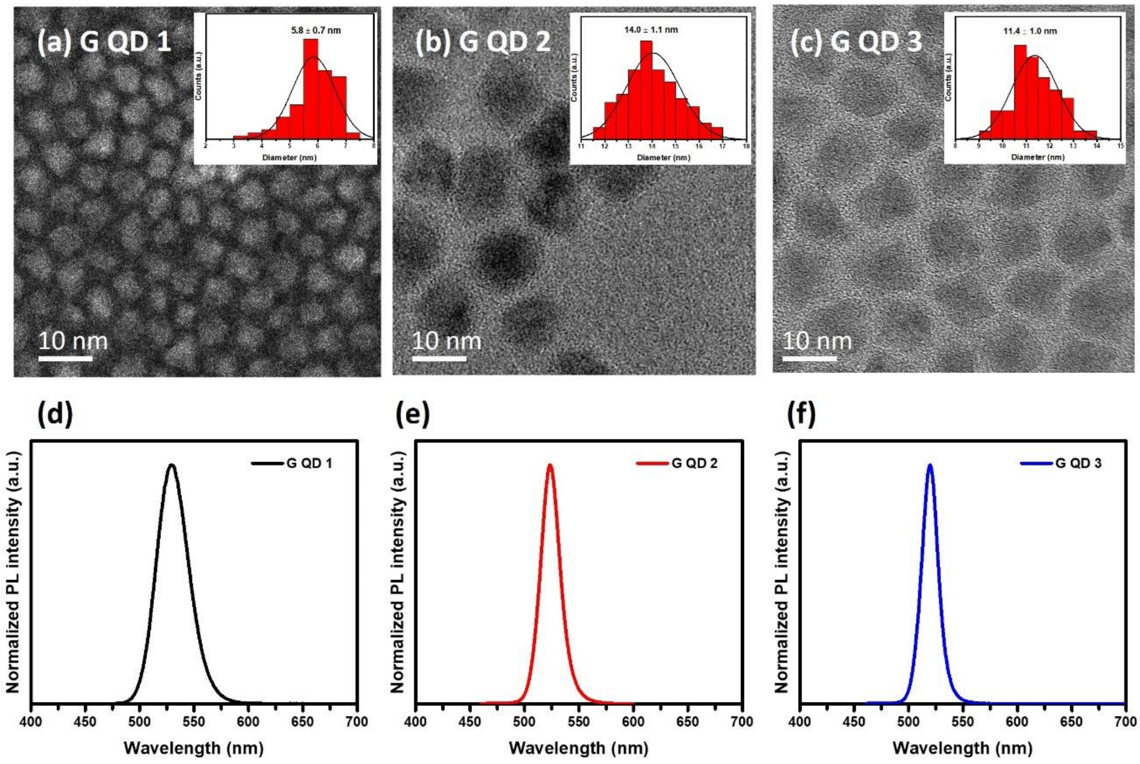

Figure S1. TEM image of dried ZnCdSeS QD film with different shell thickness (**a**) 0.5 nm, (**b**) 1.0 nm and (**c**) 1.6 nm. The size distribution are shown in the insets, they are about 5.8, 14.0, and 11.4 nm for G-QD-1, G-QD-2, and G-QD-3 respectively. (**d**)–(**f**) show the optical properties for three kinds of QDs. All of QDs are treated with the same OA ligand for fairly comparison.

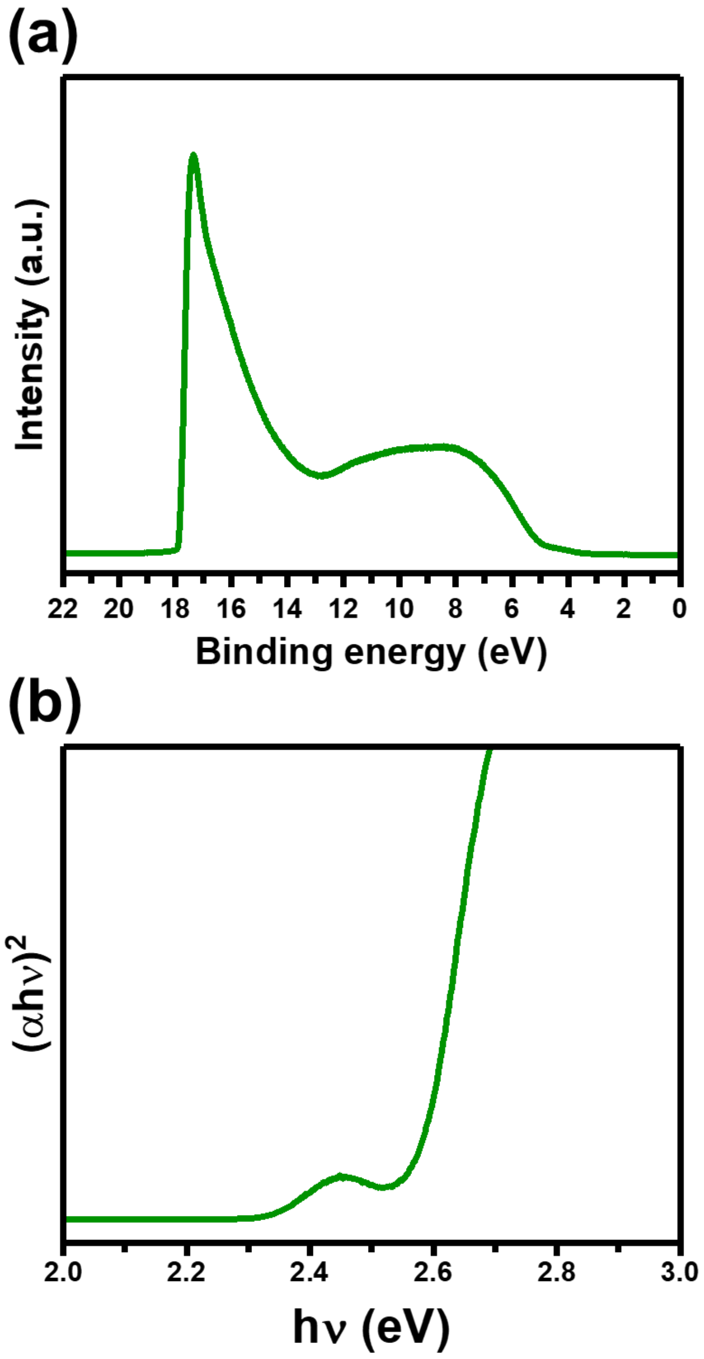

Figure S2. (**a**) UPS spectrum and (**b**) Tauc plot of the green QDs used in this work.

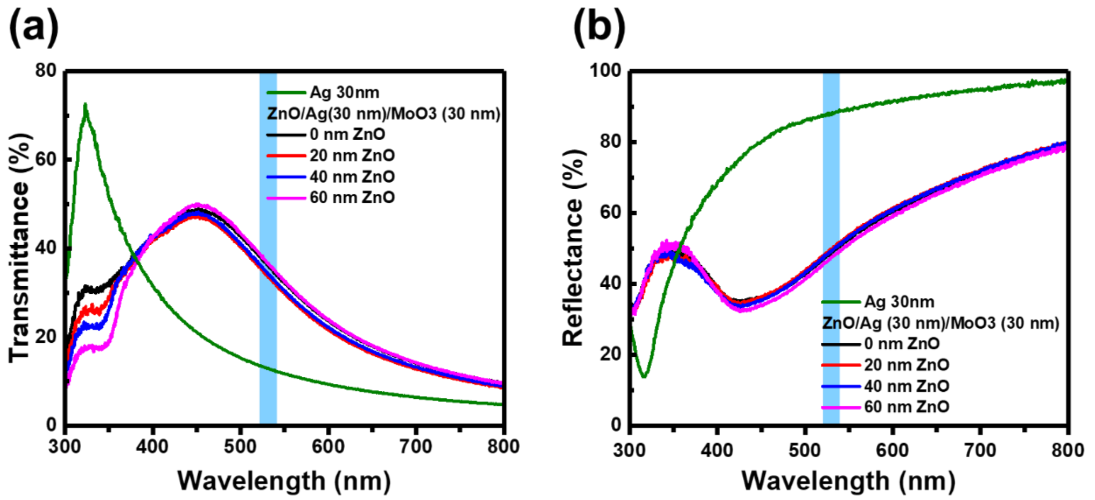

Figure S3. (**a**) The transmittance spectra and (**b**) the reflectance spectra of Ag and Ag/capping layer with different ZnO NPs thickness.

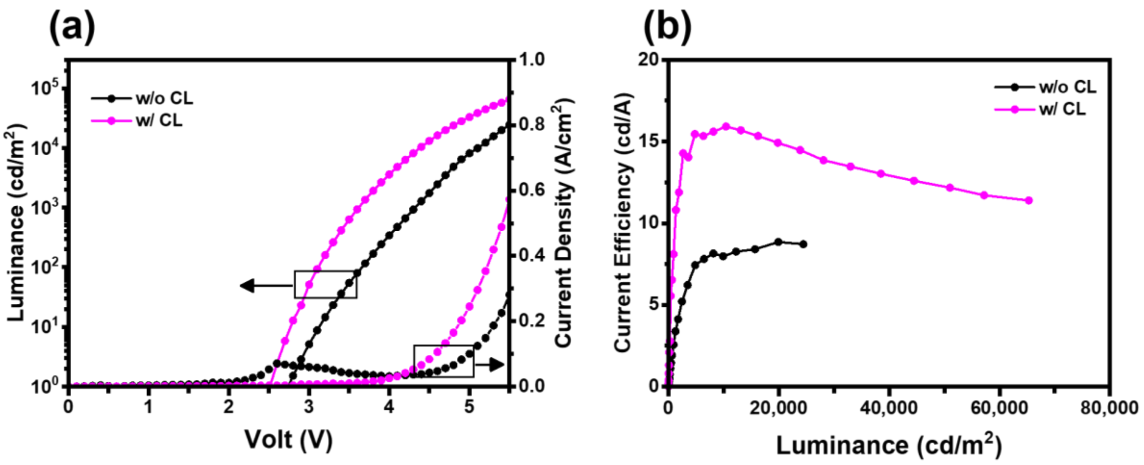

Figure S4. (**a**) Current density-voltage-luminance (*J*-*V*-*L*) characteristics and (**b**) Luminance-current efficiency characteristics for the TEQLED devices with or without a capping layer (thickness ~ 30 nm).

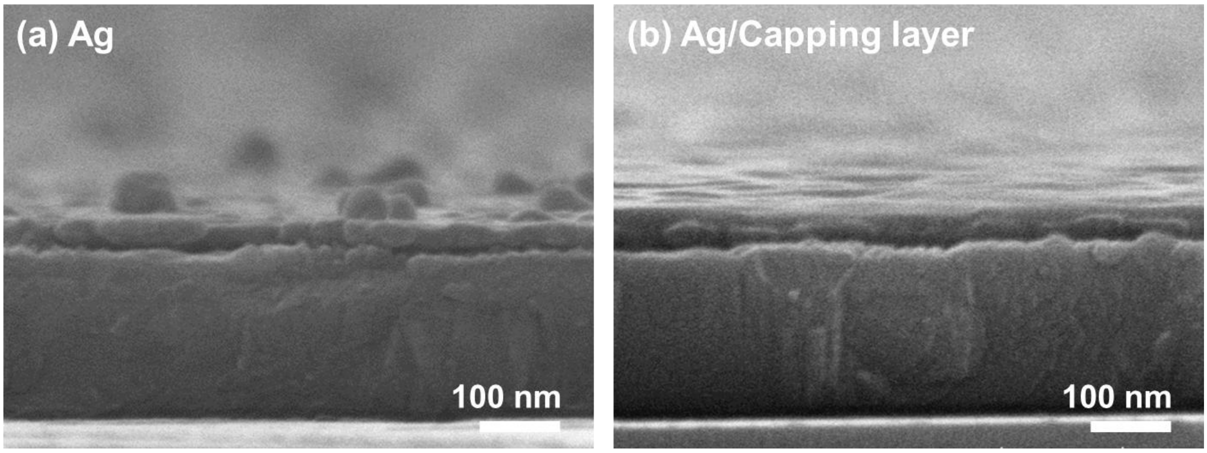

Figure S5. The cross-sectional SEM images of (**a**) Ag and (**b**) Ag/capping layer. The films are deposited on an ITO-coated glass substrate.
